# Dual-Coated Antireflective Film for Flexible and Robust Multi-Environmental Optoelectronic Applications

**DOI:** 10.3390/biomimetics9100644

**Published:** 2024-10-20

**Authors:** Hyuk Jae Jang, Jaemin Jeon, Joo Ho Yun, Iqbal Shudha Tasnim, Soyeon Han, Heeyoung Lee, Sungguk An, Seungbeom Kang, Dongyeon Kim, Young Min Song

**Affiliations:** 1 School of Electrical Engineering and Computer Science, Gwangju Institute of Science and Technology (GIST), Gwangju 61005, Republic of Korea; hjjang3472@gm.gist.ac.kr (H.J.J.);; 2 Samsung Display Co., Ltd., Yongin-si 17113, Republic of Korea; 3 School of Mechanical Engineering, Gwangju Institute of Science and Technology (GIST), Gwangju 61005, Republic of Korea; 4 Artificial Intelligence (AI) Graduate School, Gwangju Institute of Science and Technology (GIST), Gwangju 61005, Republic of Korea; 5 Department of Semiconductor Engineering, Gwangju Institute of Science and Technology (GIST), Gwangju 61005, Republic of Korea

**Keywords:** antireflection, flexible film, robust film, multifunctional film, dual coating, electronic application

## Abstract

Artificial antireflective nanostructured surfaces, inspired by moth eyes, effectively reduce optical losses at interfaces, offering significant advantages in enhancing optical performance in various optoelectronic applications, including solar cells, light-emitting diodes, and cameras. However, their limited flexibility and low surface hardness constrain their broader use. In this study, we introduce a universal antireflective film by integrating nanostructures on both sides of a thin polycarbonate film. One side was thinly coated with Al_2_O_3_ for its high hardness, enhancing surface durability while maintaining flexibility. The opposite side was coated with SiO_2_ to optimize antireflective properties, making the film suitable for diverse environments (i.e., air, water, and adhesives). This dual-coating strategy resulted in a mechanically robust and flexible antireflective film with superior optical properties in various conditions. We demonstrated the universal capabilities of our antireflective film via optical simulations and experiments with the fabricated film in different environments.

## 1. Introduction

The reduction in optical losses at interfaces between materials with differing refractive indices is critical for optimizing the optical performance of various optoelectronic fields, such as light-emitting diodes, photovoltaic devices, image sensors, cameras, transparent glasses, and energy-harvesting [1,2,3,4,5,6,7,8,9,10,11]. To address this issue, multilayer coatings have traditionally been employed as a common method for producing antireflective surfaces. These coatings are designed to minimize reflection by stacking multiple layers of materials with varying refractive indices [12,13,14]. However, while effective, multilayer coatings often face challenges related to thermal mismatch, material selection, and the complexity of optical design [14].

In recent years, artificial nanostructured surfaces, inspired by the natural design of moth eyes, cicada wings, and lotus leaves, have been developed with multifunctional nanostructures, such as antireflection, bactericidal properties, and superhydrophobicity [15,16,17,18]. Notably, artificial antireflective structures (ARSs) suppress Fresnel reflection at the interface by linearly changing the refractive index [19,20]. These antireflective nanostructured surfaces have emerged as a promising alternative with geometrical optimizations. These nanostructures effectively minimize light reflection, enhance optical performance across a wide range of wavelengths, and have a simple optical design [20]. Despite their advantages, these nanostructured surfaces encounter significant limitations, particularly in terms of flexibility, surface stability, and hardness [21,22,23,24,25]. Their limited mechanical robustness restricts their use in more demanding environments, which hinders the broader application of these antireflective surfaces in advanced optoelectronic technologies [9,10,11,26,27,28,29,30]. Overcoming these limitations is essential to fully exploit the potential of nanostructured antireflective surfaces.

In this study, we developed a universal antireflective nanostructured polycarbonate film (*uni*-ARS PC film) for flexibility with better robustness by integrating nanostructures on both sides of a thin PC film. Our proposed *uni*-ARS PC film has the advantage of reducing reflectance across various materials with different refractive indices, such as air, water, and adhesive layers, all within a single design. To enhance surface durability without compromising flexibility, one side of the film was thinly coated with aluminum oxide (Al_2_O_3_), a material known for its high hardness. The opposite side was coated with silicon dioxide (SiO_2_), which optimizes antireflective properties, making the film adaptable to different environments, including air, water, and adhesive layers.

This *uni*-ARS PC film, utilizing a dual-coating strategy, resulted in a mechanically robust and flexible antireflective film with superior optical properties across different conditions. To validate our approach, we conducted comprehensive optical simulations and performed measurements on the fabricated film in diverse environments (i.e., air, water, and display). The results demonstrate that our design effectively addresses the mechanical and optical limitations of artificial antireflective surfaces, offering a versatile solution that is suitable for a wide range of applications.

## 2. Materials and Methods

For the fabrication of the dual-coated antireflective film, a hot-pressing process was initially performed to implement ARS on a 250 μm thick PC film (LEXANTM8010, TEKRA, New Berlin, WI, USA) using a nanostructured nickel master stamp (HT-AR-02A, Temicon, Dortmund, Germany), which features a hexagonally arranged nanostructured pattern with a period of 250 nm and a height of 300 nm. The PC film was imprinted using a hot-pressing machine (QM900M, QMESYS, Uiwang-si, Republic of Korea) by pressing the film between two master molds at elevated temperatures of up to 190 °C for 5 min, with a pressure of 5 MPa for a 5 × 5 cm PC film. To ensure proper formation, cooling was then performed at 100 °C for 10 min before releasing the pressure.

Subsequently, Al_2_O_3_ was coated on the top side of the thin film using atomic layer deposition (ALD, Atomic-Classic, CN1) at 60 °C. Trimethylaluminum (TMA) and H_2_O were used as the precursor and reactant, respectively. The TMA pulse, N_2_ purge, H_2_O pulse, and N_2_ purge cycle were repeated with a base pressure of 500 mTorr and a deposition rate of approximately 0.4 nm/cycle. Finally, SiO_2_ was then deposited on the bottom side of the thin film using plasma-enhanced chemical vapor deposition (PECVD, Plasmalab 80+, OXFORD) at 150 °C for 30 s under the following conditions: RF power of 20 W, N_2_O flow of 800 sccm, and SiH_4_ of 100 sccm.

Optical performance was simulated using rigorous coupled-wave analysis (RCWA) methods to predict reflectance across various refractive indices (air, water, and adhesive) with commercial software (DiffractMod, Rsof t 2021, Synopsys, San Diego, CA, USA). The optical simulations were conducted with hexagonally arranged nanostructures, with a period of 250 nm and height in a visible wavelength. The simulations accounted for varying nanostructure heights, SiO_2_, and Al_2_O_3_ thicknesses for the coating layers to optimize the film’s antireflective properties. The optical performances of antireflective films were evaluated by the UV/visible and NIR spectrophotometer (V-770, JASCO, Easton, MD, USA) to measure total reflectance.

Mechanical robustness was evaluated through water contact angle (WCA) measurements using a contact angle analyzer (Phoenix 300, SEO, Suwon-si, Republic of Korea) before and after wear resistance tests. The wear resistance tests included rubbing cycles performed with a rubbing test machine (CT-RB1, Coretech, Incheon, Republic of Korea) equipped with a rubber stick with a durometer A-type hardness of 88 under a 1 kg load. The test films were mounted on a slide glass and positioned on a plate with a path length of 5 cm. After the wear resistance tests, both quantitative and qualitative evaluations were conducted to assess damage to the samples. Scanning electron microscopy (SEM) images were obtained using a Hitachi S-4700 SEM (Tokyo, Japan), while an X-ray photoelectron spectroscopy (XPS, NEXSA, Thermo Fisher Scientific, Waltham, MA, USA) was used to analyze surface morphology and chemical composition changes following mechanical testing.

Bending tests were performed by subjecting the films to different radii of curvature to assess crack formation and durability using a custom setup. The test films were secured on both sides using holders to evaluate the compressive stress applied to the lower side. The compressive stress on the test films was evaluated at radii of curvature of 5.5, 3.5, and 2.5 mm, respectively. Subsequently, surface damage for the bending region was examined using SEM.

The refractive index profile was calculated using numerical software (MATLAB 2016a, MathWorks, Natick, MA, USA). The average refractive index was determined by defining a unit area and calculating the area ratio based on the structure height and coating thickness. Each calculation was performed in 1 nm height increments, and a fixed refractive index value was used to simplify the calculations.

## 3. Results

### 3.1. Design and Fabrication of Dual-Coated Antireflective Film

**Figure 1:**
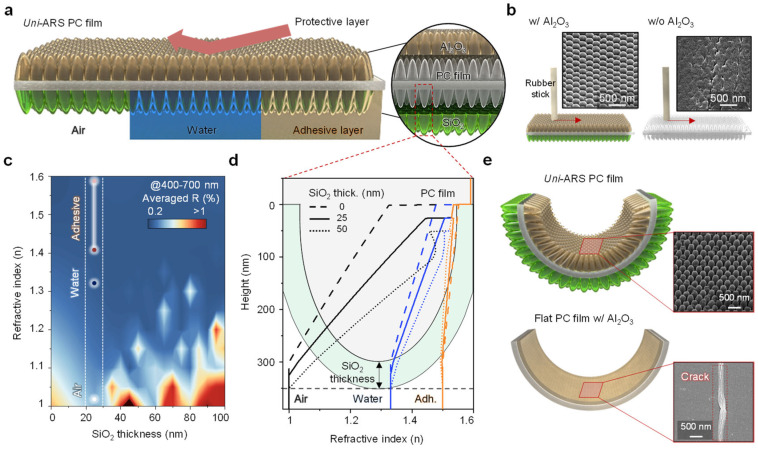
Universal design for antireflective PC film. (**a**) Schematic illustration of dual-coated antireflective film integrating nanostructures on both sides of a PC film. The top side is coated with Al_2_O_3_ to enhance surface hardness, while the bottom side is coated with SiO_2_ to optimize antireflective properties in various environments, including air, water, and adhesive layers. (**b**) A schematic illustration comparing the wear resistance with and without the Al_2_O_3_ coating. SEM images show the surface morphology, demonstrating the improved durability provided by the coating. (**c**) A reflectance contour plot showing the optical optimization between the SiO_2_ thickness and refractive index, highlighting the optimized SiO_2_ thickness for various dynamic environments, such as air, water, and adhesive layers. (**d**) Refractive index profiles, as a function of nanostructure height and SiO_2_ thickness ((**a**), dash box), illustrating how these parameters influence the antireflective performance of the film. (**e**) A schematic illustration comparing the flexibility of the film with *uni*-ARS and flat PC films. SEM images from bending tests of the *uni*-ARS PC film (top) and flat PC films with Al_2_O_3_ coating (bottom), indicating that the flat PC film exhibits cracks after the bending test, while the *uni*-ARS PC film remains crack-free.

The dual-coated antireflective film was designed by integrating nanostructures on both sides of a thin PC substrate. The film was engineered to address the limitations of traditional antireflective coatings, particularly in terms of flexibility and mechanical robustness. As illustrated in Figure 1a, the top side of the film was coated with a thin layer of Al_2_O_3_ using ALD. This coating was selected for its high hardness and ability to enhance surface durability, thereby protecting the underlying nanostructures from physical damage during use [22]. The bottom side of the film was coated with SiO_2_ via PECVD. The SiO_2_ layer was optimized to provide effective antireflective properties, making the film adaptable to various environments, including air, water, and adhesive layers.

Figure 1b presents a schematic illustration comparing the wear resistance of nanostructured PC films with and without an Al_2_O_3_ coating. SEM images show the surface morphology of the films after 5 rubbing cycles, highlighting the impact of the Al_2_O_3_ coating on wear resistance. The uncoated nanostructures exhibit significant structural degradation. By contrast, the SEM images of the Al_2_O_3_-coated film reveal that the nanostructures remain intact, indicating that the Al_2_O_3_ coating effectively preserves the structural stability of the film during the rubbing test. This evaluation is crucial in ensuring that the mechanical durability of the film is enhanced without sacrificing its optical properties.

To optimize the antireflective performance of the film, we conducted optical simulations to determine the ideal thickness of the SiO_2_ coating in relation to the refractive indices of the adjacent layers. Figure 1c presents a reflectance contour plot illustrating the relationship between SiO_2_ thickness and the refractive index. The plot demonstrates that a specific SiO_2_ thickness can minimize light reflectance across various dynamic environments (e.g., air, water, and adhesive layers). By fine-tuning the SiO_2_ thickness, the ARS PC film achieves broad-spectrum antireflective properties that are effective in multiple environments.

Additionally, the refractive index profiles, as shown in Figure 1d, were analyzed as a function of a nanostructure height of 300 nm and SiO_2_ coating thickness of 0, 25, and 50 nm. This analysis provided insights into how these parameters influence the antireflective performance of the designed film. The optimized refractive index profiles confirmed that the dual-coated design offers superior optical performance, reducing reflectance to minimal levels across different refractive indices.

The mechanical robustness of the *uni*-ARS PC film was further validated through bending tests. Figure 1e illustrates the schematic comparison of the flexibility between the *uni*-ARS PC film and the flat film. SEM images show that the nanostructured PC film coated with Al_2_O_3_ and flat PC films with the same coating were both subjected to the bending test. The flat PC film exhibited significant cracks under mechanical stress, highlighting its limited flexibility. In contrast, the nanostructured PC film remained undamaged, demonstrating that the nanostructured design, combined with the Al_2_O_3_ coating, significantly enhanced the film’s mechanical durability and flexibility.

### 3.2. Optical Performance and Simulation of the Universal Antireflective Film

**Figure 2:**
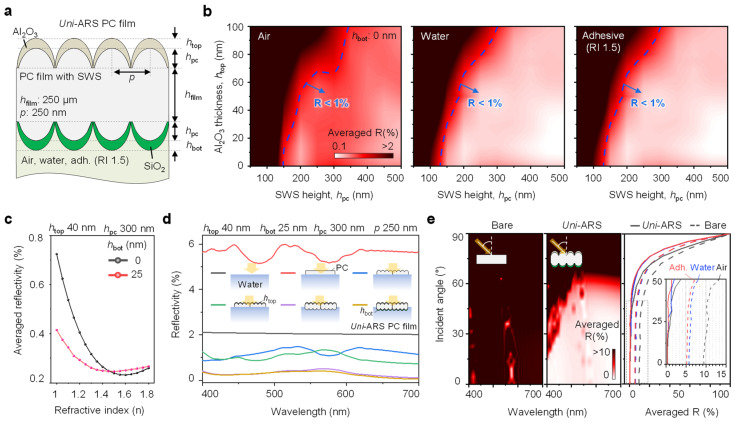
Optical simulation for the universal antireflective PC film. (**a**) A schematic of the PC film with a nanostructure for optical simulation, where the film is coated with SiO_2_ for compatibility with air, water, and adhesive environments (refractive index of 1.5). (**b**) Averaged reflectance contour plots for the film in different environments (air, water, and adhesive), showing that the reflectance is maintained below 1% across all cases with an SWS height (h_pc_) of 150 nm. (**c**) Detailed averaged reflectance profiles as a function of the bottom SiO_2_ thickness (h_bot_) for various environments, demonstrating consistent low reflectance across different refractive indices. (**d**) Schematic and reflectance profiles illustrating how the structure optimizes the optical performance of the proposed film. (**e**) Reflectance comparison as a function of the incident angle between flat and nanostructured PC films, where the flat PC film shows significantly higher reflectance compared to the nanostructured film. The right reflectance plot, according to the incident angle, confirms the consistent superior antireflective performance of the nanostructured film in air, water, and adhesive environments.

The optical performance of the dual-coated antireflective film was thoroughly investigated through both simulations and experimental measurements to assess its effectiveness across various environments with different refractive indices. Figure 2a illustrates the schematic design used for optical simulations, where the PC film is integrated with nanostructures and coated with SiO_2_ and Al_2_O_3_. The period of the nanostructures was set to 250 nm to ensure sufficient transmittance in the visible wavelength range [3]. The SiO_2_ layer was specifically tailored to enhance the film’s compatibility with environments such as air, water, and adhesive layer, which had a refractive index of 1.5. The thickness of the Al_2_O_3_ coating was optimized with reflectance and durability. To evaluate the antireflective capabilities of the film, we conducted optical simulations that show averaged reflectance contour plots for visible wavelengths of 400–700 nm, as shown in Figure 2b. The results clearly demonstrate that the film maintains a reflectance below 1% across all tested environments when the ARS height is set at 150 nm. This low reflectance is a critical feature that ensures minimal optical losses, making the film suitable for various optical applications where maintaining high transparency and minimal reflection is essential.

Further analysis was conducted to understand how the thickness of the SiO_2_ layer (h_bot_) influences reflectance profiles across different environments. Figure 2c presents the detailed averaged reflectance profiles for visible wavelengths as a function of the bottom SiO_2_ thickness. The results indicate that consistent low reflectance can be achieved across a range of refractive indices by optimizing SiO_2_ thickness. This consistency underscores the designed film’s adaptability and effectiveness in diverse applications, from air-based systems to underwater and adhesive layer environments.

To further optimize the optical performance, we examined how the structure of the nanostructured film impacts reflectance, as depicted in Figure 2d. The schematic and corresponding reflectance profiles illustrate the significance of fine-tuning the nanostructure height and SiO_2_ thickness to achieve the best possible antireflective performance. The optimized film structure demonstrates superior optical properties, ensuring that the designed film effectively reduces reflectance across multiple environments without compromising transparency.

Finally, Figure 2e compares the reflectance of flat and *uni*-ARS PC films as a function of the incident angle. The results clearly show that the flat PC film exhibits significantly higher reflectance, particularly at higher incident angles, which is undesirable in many optical applications. In contrast, the nanostructured PC film maintains a low reflectance even as the incident angle increases, confirming its superior antireflective performance. The reflectance plot on the right side further confirms the film’s consistent performance in both air and water environments, as well as when applied over adhesive layers, making it highly versatile for a wide range of optical devices.

### 3.3. Fabrication and Optical Evaluation of the Dual-Coated Antireflective Film

**Figure 3:**
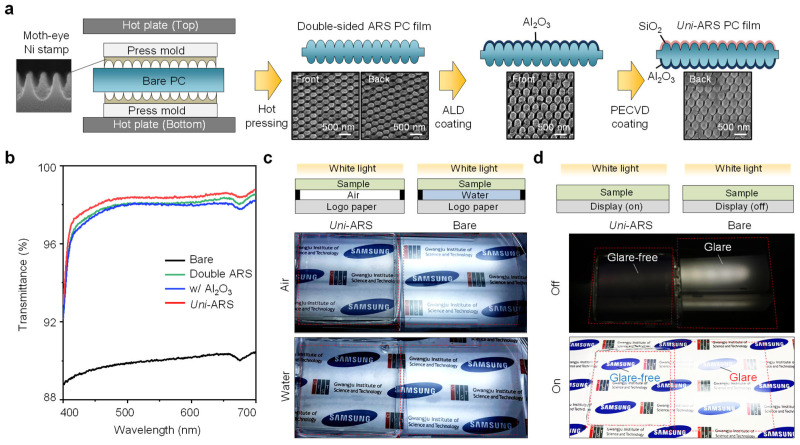
Fabrication and optical evaluation for universal film. (**a**) A schematic illustration of the fabrication process for dual-coated nanostructured PC films. SEM images showing the surface morphology of the nanostructured PC films on the front and back sides, corresponding to each fabrication process. (**b**) Transmittance spectra measured in the air environment for the fabricated film, corresponding to the process shown in Figure 3a, demonstrate that the *uni*-ARS PC film exhibits enhanced transmittance. (**c**,**d**) (**c**) Photographs demonstrating the performance of the dual-coated film under air and water conditions. The *uni*-ARS film under a white light source effectively exhibiting the underlying logo through the film in air and water. (**d**) Photographs demonstrating the performance of the dual-coated film on the display panel. The *uni*-ARS film placed over a display, showing the difference between the display being on (bottom) and off (top). The *uni*-ARS film preserves visibility by suppressing light reflection, effectively reducing glare in both display on and off conditions.

The fabrication process of the dual-coated antireflective film was carefully designed to ensure both the mechanical robustness and superior optical performance of the final film. Figure 3a shows a schematic overview of the fabrication process. The PC film was first imprinted with nanostructures on both sides using a hot-pressing technique, which involved pressing the film between two pattern molds, with a period of 250 nm and a height of 300 nm, at elevated temperatures of up to 190 °C for 5 min with a pressure of 5 MPa [31,32,33]. After imprinting, one side of the film was coated with Al_2_O_3_ at a thickness of 40 nm using ALD to enhance surface hardness and durability. The opposite side was coated with SiO_2_ with a thickness of 25 nm via PECVD, which was optimized for antireflective performance across various environments. The surface morphology of the nanostructured PC films was examined using SEM images before and after the coating processes. Those of the double-sided ARS PC film displayed the nanostructured surfaces before any coatings were applied, revealing well-defined and uniform nanostructures that are crucial for achieving low reflectance. Those of the Al_2_O_3_ and SiO_2_ coatings showed the surfaces after coating, with Al_2_O_3_ on the front side and SiO_2_ on the back side. The images confirm that the Al_2_O_3_ and SiO_2_ coatings preserved the well-defined shape of the pattern mold after the coating processes, ensuring that the optical and mechanical properties of the film were not degraded.

Figure 3b presents the transmittance spectra measured in the air environment for the fabricated *Uni*-ARS PC film, which corresponds to the fabrication process outlined in Figure 3a. This clearly shows that the *uni*-ARS PC film exhibits enhanced transmittance compared to the film without a SiO_2_ coating. This improved transmittance is attributed to a dual-coated nanostructured design, which minimizes reflection and allows for improved light transmission across a visible wavelength. This optical improvement is crucial for applications requiring minimal optical loss, making the *uni*-ARS PC film highly suitable for applications in environments where high optical clarity and low reflectance are essential, such as in displays, solar panels, and transparent electronic devices.

For qualitatively qualifying multi-environmental applications, the optical performance of the dual-coated film was further evaluated under different environmental conditions. Figure 3c presents photographs of the film tested in both air and water environments. When exposed to a white light source, the dual-coated film effectively revealed the underlying logo through the film, both in the air and when submerged in water. This demonstrates the designed film’s ability to maintain high transparency and low reflectance across different refractive indices, making it suitable for applications in dynamic environments.

Additionally, Figure 3d highlights the performance of the dual-coated film when applied to a display panel. The film was placed over the display, and its performance was assessed with the display both on and off. The photographs show that the film minimizes reflection and glare, preserving the visibility of the display content under various lighting conditions. When the display is on, the underlying text and images remain clear and distinct, demonstrating that the film effectively reduces light reflection and enhances visibility. The combination of these fabrication techniques and optical evaluations confirms that the dual-coated antireflective film offers significant advantages for both durability and optical performance. The film’s ability to adapt to different environments, along with its effectiveness at reducing reflectance, makes it a versatile solution for advanced electronic and optical applications.

### 3.4. Mechanical Performance of the Dual-Coated Antireflective Film

To ensure that the dual-coated antireflective film not only excelled in optical performance but also maintained mechanical integrity under various stress conditions, a series of mechanical tests were conducted. These tests evaluated the film’s durability, particularly its resistance to mechanical wear and deformation, which are critical factors for practical applications in flexible electronic devices.

**Figure 4:**
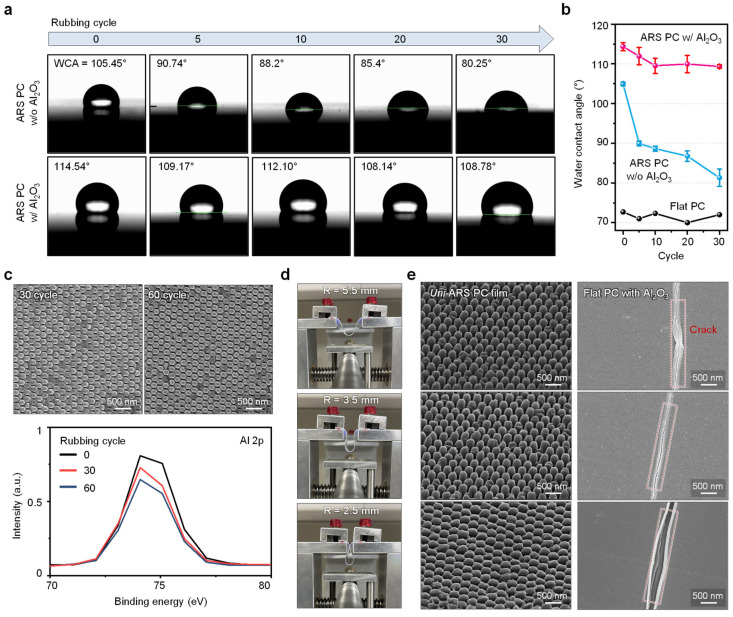
Mechanical performance of the *Uni*-ARS film. (**a**) A comparison of water contact angle measurements for the nanostructured PC films over 30 rubbing tests without the Al_2_O_3_ coating (top row) and with the Al_2_O_3_ coating (bottom row). (**b**) Graphs comparing the change in WCA for nanostructured PC films as a function of rubbing cycles in Figure 4a, indicating that the nanostructured film with the Al_2_O_3_ coating maintains its hydrophobic properties better than the nanostructured PC film without Al_2_O_3_. To evaluate the stability and reliability of the results, error bars are shown for three samples. (**c**) SEM images of the *uni*-ARS PC film after 30 and 60 rubbing cycles. The bottom plot exhibits XPS data about Al2p according to the rubbing cycle, showing durability. (**d**) Photographs of the bending test setup, showing the *uni*-ARS films under different radii of curvature (R = 5.5 mm, 3.5 mm, 2.5 mm) during the mechanical test. (**e**) SEM images comparing the *uni*-ARS PC film and flat PC film with the Al_2_O_3_ coating after the bending test, revealing cracks on the flat PC film while the *uni*-ARS PC film remained intact.

Figure 4a presents the results of the WCA measurements, which were used to assess the hydrophobicity and surface integrity of the nanostructured PC films after repeated mechanical stress. The top row shows the WCA measurements for nanostructured films without the Al_2_O_3_ coating after 30 rubbing cycles. The reduction in WCA indicates a loss of hydrophobicity and surface degradation due to mechanical stress. In contrast, the bottom row shows WCA measurements for nanostructured films with the Al_2_O_3_ coating. Even after 30 rubbing cycles, the coated film retained a high WCA, indicating that the Al_2_O_3_ coating effectively preserved the film’s hydrophobic properties and surface consistency under mechanical stress.

Figure 4b provides a graphical comparison of the change in WCA for both coated and uncoated nanostructured PC films as a function of the number of rubbing cycles shown in Figure 4a. The graph shows that while the WCA of the uncoated film decreases significantly with increasing rubbing cycles, the coated film exhibits only a minimal decrease, further confirming the protective role of the Al_2_O_3_ coating in maintaining the film’s hydrophobicity and mechanical durability. Furthermore, to evaluate the stability and reliability of the mechanical properties of the Al_2_O_3_ coatings, we measured the WCA for three samples with and without Al_2_O_3_ coatings, respectively. In all samples, the Al_2_O_3_-coated film consistently showed a higher contact angle compared to the uncoated film for overall rubbing cycles.

The durability of the film’s nanostructures under mechanical stress was further examined using SEM imaging after 30 and 60 rubbing cycles, as shown in Figure 4c. The SEM images demonstrate that the nanostructures remain largely undamaged after 30 cycles, with only minor wear observed after 60 cycles. Additionally, XPS analysis of the Al2p peak was conducted, with the results presented in the bottom plot of Figure 4c. The XPS data indicate that the Al_2_O_3_ coating remained stable and effectively bonded to the nanostructured surface even after multiple rubbing cycles, highlighting the coating’s durability.

To assess the film’s flexibility and resistance to cracking under deformation, bending tests were conducted, as illustrated in Figure 4d. The photographs show the film being stressed to different radii of curvature (R = 5.5 mm, 3.5 mm, and 2.5 mm) during the bending tests. The results reveal that the dual-coated film can withstand significant bending without visible damage or cracking, demonstrating its suitability for flexible electronic applications.

Finally, Figure 4e compares SEM images of the dual-coated nanostructured PC film with Al_2_O_3_ to those of a flat PC film with the same coating after the bending test. The flat PC film exhibits significant cracking, compromising its mechanical stability. In contrast, the designed film remains free of cracks, highlighting the superior mechanical robustness provided by the designed nanostructure and protective Al_2_O_3_ coating. This result underscores the effectiveness of the dual-coating strategy in enhancing both the optical and mechanical properties of the film, making it an ideal candidate for flexible, durable antireflective applications.

## 4. Conclusions

Moth-eye-inspired artificial antireflective nanostructured surfaces are highly effective at reducing optical losses at interfaces, offering significant benefits for enhancing optical performance in various optoelectronic applications. Despite these advantages, their broader use is constrained by limited flexibility and low surface hardness. In this study, we successfully developed a dual-coated antireflective film with multifunctional applications of flexibility and robust mechanical and optical performance, making it suitable for multi-environmental optoelectronic applications. By integrating nanostructures on both sides of a thin PC film and applying Al_2_O_3_ and SiO_2_ coatings, we achieved a *uni*-ARS PC film that consistently reduced reflectance across various refractive indices while maintaining durability under mechanical stress (e.g., compressive strain, wear resistance). The experimental results and simulations demonstrate that this advanced approach effectively addresses the limitations of traditional antireflective surfaces, offering a versatile and durable solution for advanced optical technologies.

Our *uni*-ARS PC film demonstrated remarkable performance, maintaining low reflectance across a range of refractive indices while also exhibiting superior mechanical performance, particularly with better wear resistance in flexible electronic applications. The robust and flexible nature of our film makes it an ideal candidate for use in flexible displays, solar panels, and other optoelectronic devices where both durability and optical clarity are paramount. Future research could explore a broader range of conditions and configurations to further validate the universality of the film’s performance. In addition, future studies could investigate alternative materials for the coating layers to further enhance specific properties, such as hydrophobicity or thermal stability. Furthermore, scaling up the fabrication process for industrial applications and testing the film’s performance under more extreme environmental conditions would be valuable for advanced technologies.

## Data Availability

The data that support the plots within this paper and other findings of this study are available on request from the corresponding author upon reasonable request. The data are not publicly available due to funders’ policies.
